# Genetic interaction networks: better understand to better predict

**DOI:** 10.3389/fgene.2013.00290

**Published:** 2013-12-17

**Authors:** Benjamin Boucher, Sarah Jenna

**Affiliations:** Laboratory of Integrative Genomics and Cell Signalling, Pharmaqam, Biomed, Department of Chemistry, Université du Québec à MontréalMontréal, QC, Canada

**Keywords:** genetic interaction, network, conservation, prediction, *Saccharomyces cerevisiae*, *Caenorhabditis elegans,* genomics

## Abstract

A genetic interaction (GI) between two genes generally indicates that the phenotype of a double mutant differs from what is expected from each individual mutant. In the last decade, genome scale studies of quantitative GIs were completed using mainly synthetic genetic array technology and RNA interference in yeast and *Caenorhabditis elegans*. These studies raised questions regarding the functional interpretation of GIs, the relationship of genetic and molecular interaction networks, the usefulness of GI networks to infer gene function and co-functionality, the evolutionary conservation of GI, etc. While GIs have been used for decades to dissect signaling pathways in genetic models, their functional interpretations are still not trivial. The existence of a GI between two genes does not necessarily imply that these two genes code for interacting proteins or that the two genes are even expressed in the same cell. In fact, a GI only implies that the two genes share a functional relationship. These two genes may be involved in the same biological process or pathway; or they may also be involved in compensatory pathways with unrelated apparent function. Considering the powerful opportunity to better understand gene function, genetic relationship, robustness and evolution, provided by a genome-wide mapping of GIs, several *in silico* approaches have been employed to predict GIs in unicellular and multicellular organisms. Most of these methods used weighted data integration. In this article, we will review the later knowledge acquired on GI networks in metazoans by looking more closely into their relationship with pathways, biological processes and molecular complexes but also into their modularity and organization. We will also review the different *in silico* methods developed to predict GIs and will discuss how the knowledge acquired on GI networks can be used to design predictive tools with higher performances.

## WHAT IS A GENETIC INTERACTION?

### GENERAL DEFINITION

The term genetic interaction (GI) covers a group of functional relationships between genes. One kind of these relationships, called epistasis, was first defined by Bateson and Mendel (1909). Biological epistasis was then described as the effect of one allele masking the effect of another one (Moore, 2003). Nine years later statistical epistasis, originally called “epistacy,” was described by Fisher (1919) as a significant deviation of the phenotype of a double mutant from what is expected considering the phenotypes of the single mutants.

**FIGURE 1:**
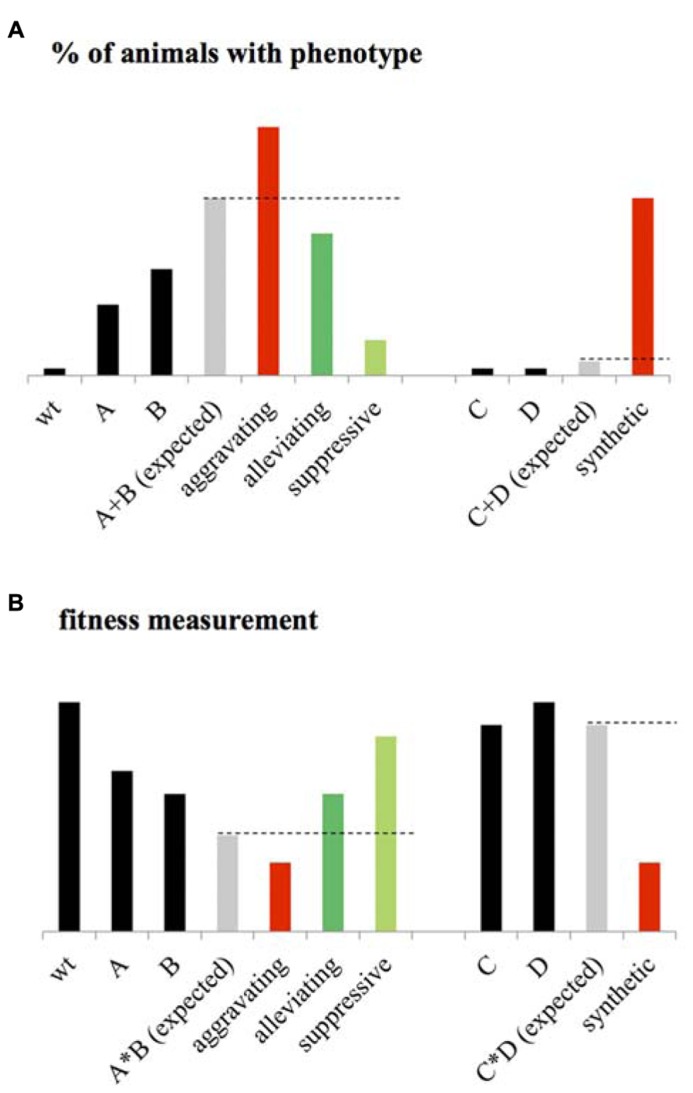
**Statistical epistasis. (A)** When considering the penetrance of a given phenotype as the percentage of animals expressing this phenotype at a given “significative” level, genetic interactions (GIs) are usually identified using the additive model. Considering the phenotype of wild-type (*wt*) animals, close to zero, the expected phenotype of the double mutant AB corresponds to the sum of the phenotypes of mutant A and B. An aggravating GI between A and B is then identified if the phenotype of AB is significantly higher than the expected. An Alleviating GI is identified if the phenotype of AB is significantly lower than expected. A suppressive interaction is identified if the phenotype of AB is lower than the single mutant with the highest penetrance. When considering two mutants C and D with no observable phenotype, a synthetic interaction is identified if the double mutant CD expresses a significant phenotype. **(B)** When fitness is measured as a phenotype, the *wt* animals present high fitness rate, the expected phenotype of the double mutant AB is calculated using the multiplicative phenotype (it could also be the Log or Min) as the product of the fitness level of A and B. An aggravating interaction is then identified if AB is significantly lower than expected. Alleviating is identified if the fitness of AB is significantly higher than expected. Suppressive interaction is identified or if the double mutant is more viable than the sickest single mutants. A synthetic interaction is identified if the double mutant presents a significant fitness defect while the two single mutants are fit.

This statistical epistasis enabled the identification of an array of different GIs. One popular classification of these GIs consists of dividing them in two main classes: the negative and the positive interactions. The negative GIs, called also aggravating or synergistic interactions, refer to an observed phenotype higher than expected when considering the phenotypes of single mutants and assuming that the mutated genes function independently one from the other (**Figure 1**). A synthetic lethal interaction, which is an extreme case of negative GI, occurs when both single mutants are viable but the double mutant is lethal (**Figure 1**). At the opposite, the positive GIs can be subdivided in buffering/alleviating interactions where the biological effect of an allele is mitigated by a second one, and also the suppressive interactions in which the double mutant is healthier than the sickest single mutant (**Figure 1**).

As mention above, identification of statistical epistasis depends on the calculation of the expected phenotype of the double mutant considering the phenotype of the single mutants and assuming a functional independency of the two mutated genes. Several models exist and are used to estimate this expected value. For developmental and population geneticists, the quantitative assessment of a phenotype involves the statistical assessment of its penetrance – the statistical occurrence of a phenotype in a group of known genotypes – considering its expressivity. A threshold is then usually set for the expressivity of the phenotype – the degree to which the phenotype expression differs among individuals – to measure the penetrance (Miko, 2008).

The development of additive, multiplicative, Min and Log models to calculate the expected phenotype of double mutants was mostly motivated by the development of systematic and large-scale screening of GIs, especially in the yeast *Saccharomyces cerevisiae* (Tong et al., 2001; Collins et al., 2007; Jasnos and Korona, 2007; Costanzo et al., 2010). These studies identified GIs based on fitness measurements (**Figure 1B**), a class of phenotype that is measured in terms of population allele frequency (Wolf et al., 2000; Otto and Lenormand, 2002; Puniyani et al., 2004), growth rate, or number of progeny of mutant strain relative to wild-type (Elena and Lenski, 1997; Szafraniec et al., 2003; Segre et al., 2005; Sanjuan and Elena, 2006; St Onge et al., 2007). The additive and multiplicative models, originally used by developmental geneticists (**Figure 1A**) and fitness measurements in yeast (**Figure 1B**) respectively, consider the expected phenotype of a double mutant to be the sum (or the product) of the phenotypes measured for the single mutants if the two mutated genes function independently one from the other (Mani et al., 2008). The Log model has been specifically designed to identify GIs from measurements on a logarithmic fitness scale (Mani et al., 2008). The Min model considers that for non-interacting genes, the fitness of the double mutant should be similar to the fitness of the less-fit single mutant. Although these models agree under certain circumstances, they often diverge dramatically (Mani et al., 2008). For example, while the Min model appears to be highly suitable for pairs of genes with more extreme single-mutant defects, this model is clearly not ideal for defining alleviating interactions and more particularly, several epistatic interactions for which a double mutant phenotype is similar to that of the single mutant with the most severe phenotype (St Onge et al., 2007). Unfortunately, GIs identified using this model account for a large part of all GIs found in interaction databases. This tends to bias the yeast genetic interactome against this later kind of GIs (Mani et al., 2008). Identification of GIs considering several of these models would then be an appropriate approach to enable fair comparison and integration of GIs from different screening pipeline into a homogeneous GI interactome.

### LEVELS OF ABSTRACTION IN BIOLOGICAL SYSTEMS

Mapping of GI networks is an endeavor that attracted more attention with the emergence of network and systems biology approaches. Network biology consists in simplifying complex biological systems into different layers of graphical representations in which nodes correspond to physical elements (genes, protein, metabolites, RNA, etc.) and edges refer to different relationships between these elements. Systems biology, and more particularly integrative genomics, aims to better understand the structure and the functioning of the system through integration of these different networks (Ge et al., 2003).

**FIGURE 2:**
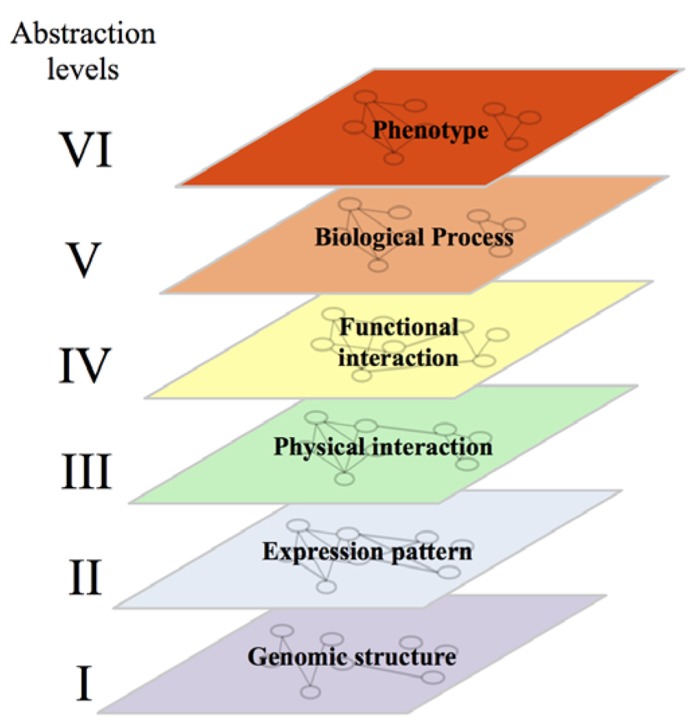
**Representation of the six levels of abstraction in biological systems.** Note that, while each gene/protein can be followed from one abstraction level to another, the relationships linking it with its neighbors are different at each level. The conservation of links between two levels of abstraction in a given system and between orthologous genes/proteins in different systems are discussed in the main text of this review.

In computer sciences, organization of systems into several abstraction levels aims to hide a certain level of detail to allow the programmer to focus on a given problem. For a computer, the lower level of abstraction would contain details on the hardware while the higher level will represent the logic of the program. In agreement with this approach, a systems biologist will consider a biological system with all its complexity and identify, from the genomic sequence to the phenotype, different levels of abstractions. At the lower level of this conceptual structure, we would find several networks representing the physical structure and organization of the genome. In these networks, nodes could be genes/coding sequences, single-nucleotide polymorphisms (SNPs) or coding sequences linked by edges representing their physical proximity and organization within chromosomes, their homology etc. (**Figure 2**, level I). The second level of abstraction would represent the expression of that genome into physical components: proteins and RNA. Edges between these elements would indicate that they are co-expressed in different contexts or that their expression profiles throughout multiple experimental conditions are highly correlated (**Figure 2**, level II; Ge et al., 2003; Vidal et al., 2011). The third level of abstraction would represent physical interactions between different elements – protein–protein (PPI), protein-DNA (PDI) or protein-RNA (PRI) interactions (**Figure 2**, level III; Vidal et al., 2011). The fourth level of abstraction will allow the visualization of the functional relationships linking these physical elements. This level would contain GI networks, signaling and metabolic pathways (**Figure 2**, level IV). The fifth level would represent biological processes. This level would contain networks where proteins implicated in the same biological process would be linked by an edge (**Figure 2**, level V). The sixth and last level of abstraction would represent phenotypes and show the relationships between elements associated with similar phenotypes and diseases (**Figure 2**, level VI). Breaking down through the different levels of abstraction aims to understand the molecular basis of higher levels. A huge amount of effort is being made to enable such a breaking down and to establish the links and the dynamics underlying the relationships between networks located at the different levels. The relationship between the second (gene expression) and the third level (mainly PPI and PDI) has been well documented. Some studies showed that interacting proteins are more likely to be encoded by genes with similar expression profiles than non-interacting proteins (Ge et al., 2001; Grigoriev, 2001; Mrowka et al., 2001; Jansen et al., 2002; Kemmeren et al., 2002). Similarly, expression profiles can be used to understand the organization and dynamics of protein interaction networks through functional characterisation of highly connected nodes (Hubs). For example, Hubs have been divided into “party” and “dating” Hubs. The former class of Hubs corresponds to proteins that tend to be co-expressed with their protein partners while the later ones are not (Han et al., 2004). Party Hubs have then been proposed to interact with all their protein partners in all biological conditions, while dating Hubs may interact with subgroups of their protein partners in certain conditions and/or environments (Han et al., 2004). PPIs and PDIs can also be used to understand the molecular basis of co-expression (Lee et al., 2002; Segal et al., 2003; Yu et al., 2003; Luscombe et al., 2004).

The link between the third (molecular interactions) and the fourth level (functional interactions) has also been investigated. Notably, signaling and metabolic pathways were shown to be enriched in PPIs and PDIs (Vidal et al., 2011). It is important to notice that, as detailed in the third chapter of this review, the term pathway has been assimilated in several papers as PPI and PDI modules – PPI/PDI subnetworks with a high density of links – or as dense GI network structures (Kelley and Ideker, 2005; Bellay et al., 2011a). Here, signaling and metabolic pathways will be described as a group of molecules functioning together and most of the time, in cascade to control a biological function. As detailed in the following chapters, GI networks are also linked to PPI and PDI networks (see *In Silico* Mapping of GIs). This link is however less evident than the link between PPI/PDI networks and signaling/metabolic pathways (see *In Silico* Mapping of GIs).

The relationship existing between the level six (phenotypes and diseases) and the level four (functional interactions) motivated the construction of pathway databases such as Reactome (Joshi-Tope et al., 2005) or the kyoto encyclopedia of genes and genomes (KEGG; Kanehisa and Goto, 2000), and is at the forefront of the research effort to identify therapeutic targets and pharmaceutical compounds (Yuryev, 2012).

The link between the levels four (functional interactions) and five (biological processes) is clear for signaling and metabolic pathways. Each signaling pathway, for example the EGF receptor/Ras/MAP kinase pathway, involves proteins that can be grouped based on their implication in the control of various biological processes, e.g., endocytosis, Ras regulation, actin cytoskeleton remodeling, kinase activity/phosphorylation, etc.

Abstractions levels can also be linked to distant levels. For example, GIs are shown to be enriched in co-expressed genes (Zhong and Sternberg, 2006; Lee et al., 2010a; link between the fourth and the second level). Similarly, integration of the sixth level (phenotype) to the third (PPI) permitted the construction of the human disease interactome. This interactome was proposed to support the existence of disease specific functional modules and also to help the molecular characterization of the protein products of disease genes (Goh et al., 2007).

Integration of different networks within or across abstraction levels brings substantial information on the structure of the system, and to some extent, information about its dynamics (Han et al., 2004). These pieces of information constitute, as described in this review, the baseline for the construction of predictive tools used to enrich and complete sparse networks.

We will focus, in this review, on the fourth level and more particularly, on GI networks. While this kind of functional relationship is linked to higher and lower levels of abstraction, most of these links appear much less clear than those involving signaling and metabolic pathways. We can then wonder if mapping such a network is of biological interest: would it bring complementary information to those brought from pathways dissection and significantly help understanding the functioning of the system?

### WHY CONSTRUCTING A CATALOG OF GENETIC INTERACTIONS?

There are two main reasons why mapping GI networks is of biological interest. The first one is to understand the mechanisms underlying the robustness of biological systems. How the system compensate for the loss or alteration of a biological function or the alteration of its environment?

Unnecessary genes do not exist in biological systems and would be eliminated through evolutionary processes (Stern and Orgogozo, 2009). So, why 73% of these necessary genes appears not to be essential (Giaever et al., 2002)? Because compensatory relationships exist between genes, pathways, and biological processes. Therefore, mapping of GIs appears to be the best way to identify these compensatory phenomena. In addition to the high contribution this mapping will bring to basic sciences, it is also of high interest for translational research. Biological robustness is indeed, a major problem in the pharmaceutical industry with the development of resistance to therapeutic agents, particularly to anti-cancer chemotherapies (Edelman et al., 2010). Identification of compensatory relationships between genes and pathways, through mapping of GIs, appears then as an avenue that needs to be explored in parallel with the dissection of the pathways themselves.

The second reason is associated with the still mysterious relationship existing between genotype and phenotype. Population geneticists highlighted the intricate complexity of genetic variations and how positive and negative relationships between alleles influence phenotypical outcome (Gibson, 2010). Cancer modifier loci, including “susceptibility” or “resistance” alleles, are good examples of genetic variations affecting a patient phenotype, here the aggressiveness of the tumor phenotype (Dragani, 2003). Similarly, GIs and more particularly digenic synthetic GIs may underlie many common diseases that are familial but not Mendelian in their inheritance, such as glaucoma, type II diabetes, lupus erythematosus and schizophrenia (Tong et al., 2004). Exploring GI networks in model organisms, through screening of low order (between two alleles) and high-order interactions (between more than two alleles), may then help understanding the genetic networks underlying phenotypical variations and multigenic diseases (Lehner, 2011).

## MAPPING GENETIC INTERACTOMES IN MODEL ORGANISMS

### IN YEAST

Quantitative studies of synthetic sick or lethal (SSL) interactions in the baker’s yeast *S. cerevisiae* represent most of the GIs screens done to date. The existence of mutation libraries for both essential and non-essential genes is regarded as the main reason for the development of large-scale GI studies (Giaever et al., 2002). Non-essential gene mutant libraries contain strains where single gene coding sequences are substituted by a drug-resistance marker (Giaever et al., 2002) while essential genes mutant libraries consist in a collection of conditional alleles (Tong et al., 2001; Davierwala et al., 2005; Schuldiner et al., 2005; Costanzo et al., 2010). These libraries have been extensively used in an automated methodology called synthetic genetic array (SGA; Tong et al., 2001, 2004). SGA screening consists in using single mutated yeasts as query against a whole deletion library for the construction of double mutants in a high-throughput fashion (Tong et al., 2001, 2004). The fitness defects of double mutants are then scored to uncover SSL interactions for non-essential genes (Tong et al., 2004; Sharifpoor et al., 2012) and essential genes (Tong et al., 2001; Davierwala et al., 2005; Schuldiner et al., 2005; Costanzo et al., 2010).

In parallel, the epistatic mini-array profile (E-MAP) – another variant of SGA – takes colony size measurements (based on imaging) as a basis for the detection of GIs (Schuldiner et al., 2005). GIs are then identified through measurement of a slower (SSL, negative GIs) or faster (alleviating, positive GIs) growth rate of the double mutants than what is expected from each single mutant growth rate. This allowed the identification of both positive and negative GIs while SGA was set originally to detect negative SSL GIs only. E-MAP was also used to map GIs in different yeast species such as *Schizosaccharomyces pombe* (Ryan et al., 2012).

Among the other high-throughput methods to discover GIs in yeast, diploid-based synthetic lethality analysis with microarrays (dSLAM), uses a library of barcoded mutants and barcode microarrays to measure the relative abundance of each barcoded double mutants in pooled populations to identify digenic SSL interactions (Pan et al., 2006; Lin et al., 2008). Optical density measurements (St Onge et al., 2007), biomass quantification analysis termed flux balance analysis (FBA) (Segre et al., 2005), quantitative phenotype (Drees et al., 2005) and gene expression data (Van Driessche et al., 2005) have also been employed to map GIs in specific biological processes. However, these studies remain restricted in terms of genome coverage.

### IN* C. elegans*

Screening a large amount of GIs in the nematode requires the utilization of RNA interference (RNAi) through soaking animals in a solution containing RNAi molecules or feeding them with *E. coli* strains expressing the RNAi (Maeda et al., 2001; Timmons et al., 2001). This approach induces a downregulation of the expression of targeted gene, instead of a deletion. This has to be taken into consideration when comparing the *Caenorhabditis elegans* and yeast genetic interactomes (Lehner, 2007; Dixon et al., 2009). To identify a GI, either both genes are targeted using RNAi or a genetic mutant strain containing either a hypomorphic or a null allele can be submitted to RNAi targeting the other gene (Kamath et al., 2003; Lehner et al., 2006; Byrne et al., 2007). Both approaches have been used to map a quite limited area of the *C. elegans* genetic interactome (<2,000 GIs) when compared to genetic studies in yeast (>200,000 GIs; Lehner et al., 2006; Byrne et al., 2007; Tischler et al., 2008; Costanzo et al., 2010).

### IN HUMAN

To identify GIs in human, apart from the RNAi treatment of specifically mutated cell lines (reviewed in Dixon et al., 2009), Lin et al. (2010) suggested an interesting method that uses radiation hybrid (RH) genotyping data sets. This approach, while being fast and inexpensive, is different than standard RNAi screening in that RH panels are used in order to “simulate” a double mutations. The simulation is done with medium-selected cells that possess extra copies of two genes and “attractive” or “repulsive” interactions are then identified whether the promoting effect of the extra copies is death or survival of the cell line respectively. The results obtained using this approach could not be easily compared to negative and positive interactions observed through gene deletion and/or expression reduction. By joining several data sets of RH panels, a network of ~6.7 million potential GIs were extracted and covered ~3.4% of all human gene pairs (Lin et al., 2010).

## *IN SILICO* MAPPING OF GIS

Only few organisms, mainly unicellular, are amenable to an experimental mapping of GIs through genome-wide screening. Mapping of genetic interactomes in higher organisms requires development of predictive tools that allow a significant reduction of the number of gene pairs to be tested experimentally.

During the last decade, numerous strategies have been used to infer GIs in unicellular and multicellular organisms (**Table 1**; reviewed in Steen, 2012). However, to date, only *S. cerevisiae* and *C. elegans* genetic networks have gained substantial information from large-scale machine learning studies. Numbers of tools were developed to predict PPIs, co-essentiality, genes with similar functions, genes functioning in the same molecular complex and GIs. The design of these tools highlighted the intimate link existing between different networks – GI networks being used to infer PPIs and co-functionality (Tong et al., 2004; Ye et al., 2005a) and inversely PPI networks, phenotypic profiles and GO annotations being used to predict GIs as detailed below. These different predictors present also cross-specificities – GIs occurring to some extend between genes coding for interacting or non-interacting proteins, between or within-pathways/molecular modules, between genes involved in the same biological process or being involved in different and compensatory processes as discussed below.

**Table 1:** *In silico *methodologies for the predictions of genetic interactions.

Reference	Type	Data	Training	Method	Number of predictive features	Experimental validation	Note
Tong et al. (2004)	Yeast SSL	Genetic interactions (GI)	~4,000 GIs	Network connectivity	1	No	GIs for ~20% of query genes
Wong et al. (2004)	Yeast SSL	Protein function and localization, gene expression, protein–protein interactions (PPI), phenotype, sequence homology	795,732 gene pairs (incl. 4,598 SSL)	Decision tree	26	Yes	Network topology and “2hop” relationships
Kelley and Ideker (2005)	Yeast SSL	PPI, protein-DNA, protein-reaction	4,849 SSLs and 27,604 PPIs for “naïve predictions”	Network connectivity	2	No	Between-pathway and within-pathway models
Zhong and Sternberg (2006)	*C. elegans* GIs	GIs/PPIs orthologs from yeast/fly; anatomical expression, phenotype, GO term, mRNA co-expression along with with orthologs (yeast/fly)	1,816 GIs; 2,878 PPIs; 3,296 *cis* markers as negatives	Logistic regression	5	Yes	18,183 high-confidence GIs covering 2,254 genes
Qi et al. (2008)	Yeast SSL	GIs	13,022 SSL	Graph diffusion kernel	1	Yes	Non-restricting distance measures to find new interacting partners
Paladugu et al. (2008)	Yeast SSL	PPIs	6,074 SSL; 400,473 negatives	Support vector machine	13	No	Graph-theoretic features using only PPIs as a network
Chipman and Singh (2009)	SSL for *S. cerevisiae* and *C. elegans*	Yeast: GO, PPI; worm: PPI with yeast/human orthologs, gene expression	Yeast: 22,432 SSL and 726,457 negatives; worm: 3,863 SSL and 58,579 negatives	Decision tree	5–6	No	Random walks algorithm to achieve topological similarity measurements between gene pairs
Lee et al. (2010b)	*C. elegans* GIs	mRNA co-expression, PPI, cocitation of genes name, phylogenetic profile analysis	626,342 GO-annotated gene pairs	Weighted sum	21	Yes	Functional network-guided predictions of genetic modifiers
Lee et al. (2010a)	*C. elegans* GIs	mRNA co-expression, PPI, phenotype	1,522 GIs	Logistic regression	6	Yes	Extended multi-species interactome and new phenotype/PPI network-based features
Szappanos et al. (2011)	*S. cerevisiae GI degree*	*GIs*	3,572 SSL and 1,901 positive GIs	Flux balance analysis	1	Yes	Prediction of GI connectivity for metabolic genes
Koch et al. (2012)	*S. cerevisiae GI degree*	*Several types including mRNA expression, GO, PPI, copy number, phylogenetic profile analysis*	~3,500 GIs with 63.2% of all genes used in training	Decision tree	16	Yes	Prediction of GI degrees in *S. pombe*
Hoehndorf et al. (2013)	Fish, yeast, fly, worm, mouse GIs	GO, GI, PPI, phenotype	GIs and PPIs as positives	Semantic similarity (with the Jaccard index)	2	No	Prediction of GIs from infererred gene functions

Intuitively, we expect that the GI world constitutes a patchwork of functional relationships with distinctive properties. Predictive tools capturing different properties will then be able to identify a portion of the GI interactome and will be complementary one to another. Ultimately, acquiring a good knowledge on the molecular particularities of subclasses of GIs will lead to the design of specific and accurate predictors. To make an informed choice on the different elements that could be employed to design these predictors, we will review here the different structural and functional particularities of GIs, and detail how they have been used or could be used to generate predictor for GIs.

### EXPLOITING THE PROTEIN–PROTEIN AND GENETIC INTERACTION NETWORK DENSITY AND STRUCTURE

A primary attribute of biological interaction networks, including GI networks, is a scale-free/power law distribution of connections, where most nodes are sparsely connected (“non-Hub” nodes) and few ones are highly connected (“Hub” nodes) (Watts and Strogatz, 1998; Jeong et al., 2001; Wagner, 2001; Tong et al., 2004). These networks appear also to exhibit a small-world organization – dense interacting modules are sparsely connected to other modules but with a short average path length (Watts and Strogatz, 1998; Jeong et al., 2001; Wagner, 2001).

There is a clear connection between PPI- and GI-Hubs since a protein with many interactions in the physical network (PPI-Hub) typically has also many interactions in the genetic network (GI-Hub; Ozier et al., 2003; Kafri et al., 2008). Both kinds of Hubs tend to be essential or associated with severe fitness defects, and to genetically interact with each other (Ozier et al., 2003; Davierwala et al., 2005; Lehner et al., 2006; Goh et al., 2007; Baryshnikova et al., 2010; Costanzo et al., 2010; Sharifpoor et al., 2012). Intuitively, we may see essential Hubs as a direct association with human diseases. However, it is important to notice that, while PPI-Hubs tend to be ubiquitously expressed, disease genes (such as inherited disease genes) tend to encode for PPI-non-Hubs and to be tissue specific (Goh et al., 2007; Vidal et al., 2011).

Comparative analysis of the yeast interactome networks also revealed that the “non-essential” SSL network is at least four times denser than the PPI network (Tong et al., 2004), while the “essential“ SSL network is five times denser than the “non-essential” SSL (Tong et al., 2001, 2004; Davierwala et al., 2005). The higher density of essential when compared to non-essential GI networks, suggests that essential genes are highly connected Hubs within GI networks, and that essential pathways may be connected to number of compensatory pathways (Davierwala et al., 2005; Costanzo et al., 2010). Given that 18% of all yeast genes are essential (Giaever et al., 2002; Christie et al., 2004), this also suggests that most yeast GIs may involve at least one essential gene (Davierwala et al., 2005). The higher density of GI network, when compared to PPI network, may reflect the fact that in the case of two compensatory pathways, PPIs may occur between proteins of a linear pathway, while any member of each pathway may genetically interact with any component of its own pathway or of its compensatory pathway (Tong et al., 2004).

As shown for PPI networks, the interaction density is not homogenously distributed within GI networks that are composed of dense modules (Tong et al., 2004). These structures, as detailed above and in the following sections, are enriched in interactions occurring within functional modules (such as signaling pathways or protein complexes) or between functional modules. This property of dense GI modules could directly be used to predict novel GIs within a non-saturated network. Tong et al. (2004) showed for three specific GI modules, that ~20% of genes that interact with a high number of common partners – being part of the same dense GI module – also genetically interact one with the others. This was significantly higher than what was measured in random networks (approximately 1%; Tong et al., 2004). Qi et al. (2008) extended this network analysis by including neighbors of interacting genes from any distances and by classifying those distances by the parity of the path lengths. They employed a graph diffusion kernel that uses weighted sums for different path lengths and found that odd-length kernels were better at predicting GIs while even-length kernels were more effective in finding new PPI partners (Qi et al., 2008).

Several methods have been developed to dissect complex networks into functionally meaningful modules. Using various clustering techniques, some studies reordered the GI matrix to sort genes according to the similarity of their GI profiles. Congruent genes are then defined as genes with similar GI profiles (Schuldiner et al., 2005; Ye et al., 2005b; Collins et al., 2007; Costanzo et al., 2010, 2011). The resulting map has a modular structure that distinguishes between major biological processes, such as transcription and chromatin remodeling, DNA replication and repair or sister chromatid segregation. These GI profiles then provide a powerful way to identify sets of genes functioning in the same biological process (Tong et al., 2004; Schuldiner et al., 2005; Ye et al., 2005b; Pan et al., 2006). Some of these methods have used the complex and pathway (COP) scores for finding sets of genes that are both highly correlated and that lack an aggravating GI (Schuldiner et al., 2005; Collins et al., 2006, 2007). The top-scoring gene pairs using this method included several sets of known complex or linear pathway components, as well as several predictions of novel ones (Schuldiner et al., 2005). Mutual clustering coefficient (MCC) was also employed to measure the neighborhood sharing of connections in the GI network – called congruence score (Ye et al., 2005a,b). A high score indicates that two genes share more GI partners than expected by chance. The resulting scores are then used as weight for non-directed edges linking genes within a congruence network (Ye et al., 2005b). By comparing path lengths in three types of networks (GI, genetic congruence, and protein interaction), they showed that high genetic congruence exhibits correlation with direct PPI linkage and also exhibits proportionate distance with the PPI network (Ye et al., 2005b). This congruent score can then be used to predict PPIs.

Altogether, these studies showed that the structure of the GI network contains enough information to predict novel GIs and also to predict novel PPI, highlighting the intricate relationship existing between PPI and GI networks.

By further exploiting the relationship between PPI and GI networks, Paladugu et al. (2008) showed that PPI network graph-theory properties could also be used to predict GIs. They showed that proteins coded by SSL gene pairs, as compared to non-SSL ones, tend to have higher average degree, closeness centrality, information centrality and number of mutual neighbors within PPI network (Paladugu et al., 2008). When combined, these graph-theory properties of PPI network provided a powerful tool to predict SSL GIs (Paladugu et al., 2008). Moreover, this approach showed that the PPI network alone contains enough valuable information to predict SSL interactions. This approach appears particularly useful to predict GIs in higher organisms which are hardly amenable to systematic screening of GIs while having their PPIs at least partially mapped.

**FIGURE 3:**
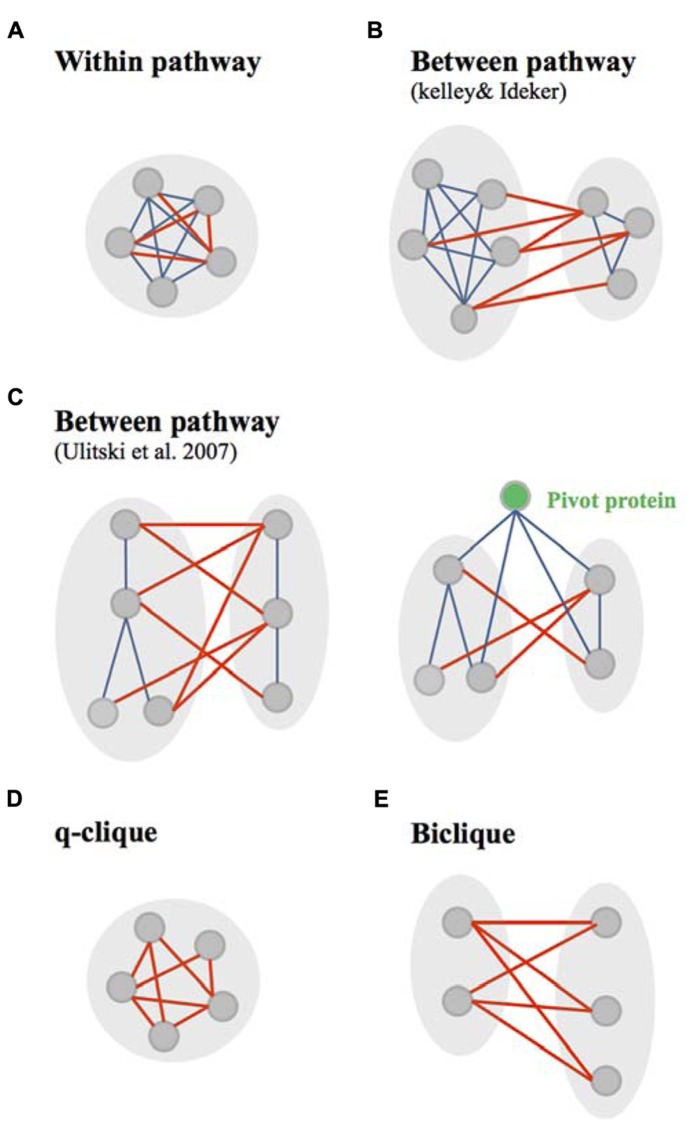
**Canonical and non-canonical within/between pathway models. (A)** The canonical within pathway model as described by Kelley and Ideker, consists in genetic interactions (GIs, red edges) occurring within a dense module of protein interactions (bleu edges). **(B)** The canonical between pathway models, as described by Kelley and Ideker (2005) , consists in GIs occurring between dense modules of protein interactions. **(C)** The canonical between pathway models, as defined by Ulitsky et al., 2008, consist in GIs occurring between connected subnetworks/graph modules of protein interactions. This study also identified pivot proteins as proteins highly connected at the molecular level with component of two subnetworks connected through between pathway GIs. **(D)** Non-canonical within pathway model, are quasi-cliques (q-cliques), biclusters of highly connected genes. **(E)** The non-canonical between pathway models consist in bicliques – biclusters in which prays and target genes of GIs do not overlap.

Few methods used GI and PPI networks to observe the distribution of GIs within or between dense modules of physical interactions (PPI and PDI), called in these studies “pathways” (**Figures 3A,B**; Kelley and Ideker, 2005; Ulitsky and Shamir, 2007). Canonical “within and between pathway models” were originally identified by Kelley and Ideker (2005). They found that the “between pathway model,” consisting of GIs occurring between dense modules of molecular interactions (**Figure 3B**), can explain three-and-a-half times as many GIs as the “within pathway” involving GIs within dense molecular interaction modules (**Figure 3A**; Kelley and Ideker, 2005). Further arguments for the prevalence of between-pathway GIs in yeast were given by Ye et al. (2005a) and Pan et al. (2006) who postulated that genes in the same pathway are expected to share common GI partners. The between and within pathway models were however shown to explain only 40% of all yeast GIs (Kelley and Ideker, 2005). Ulitsky and Shamir (2007) extended this interactome coverage by defining “pathways” as connected subnetworks within the physical interaction network rather than a dense interaction module (**Figure 3C**). This study provided a significant increase from the number of interactions explained by the Kelley and Ideker models (Ulitsky and Shamir, 2007).

Kelley and Ideker (2005) used their within and between pathway models to predict novel GIs. A five-fold cross validation technique was used to investigate the accuracy of predicting GIs using both the “within pathway model” – genes within a given pathway genetically interact more frequently than expected by chance – or using the “between pathway model” – genes in one pathway genetically interact with many of the same partners in a second pathway. They showed that both models are efficient for predicting GIs while the “between-pathway” model appears to outperform the “within-pathway model” (Kelley and Ideker, 2005).

Deeper studies on the “between and within pathways models” showed that they were often monochromatic, meaning that they were composed almost exclusively of a single type of GIs, either all negatives or all positives (Segre et al., 2005; Costanzo et al., 2010; Michaut et al., 2011). Monochromatic patterns have been used to identify biological processes and other functional modules (Segre et al., 2005; Pu et al., 2008; Jaimovich et al., 2010). Monochromatic processes are functionally diverse, but also biased (Michaut et al., 2011; Szappanos et al., 2011). For instance, microautophagy and histone exchange are monochromatic positives whereas protein import and small GTPase mediated signal transduction are monochromatic negatives (Michaut et al., 2011). Importantly, those studies showed that protein complexes are often monochromatic (Bandyopadhyay et al., 2008; Costanzo et al., 2010) and that monochromatic patterns, identified within and between biological processes, are mainly dependant on protein complexes (Michaut et al., 2011). The distinction between negative and positive interactions, when considering the relationship between PPIs and GIs, has not yet been exploited to predict GIs to the best of our knowledge. The monochromaticity and the functional bias of this monochromaticity pattern have not been exploited neither.

In contrast to what was shown in yeast, the “within pathway model” tends to be more prevalent when compared to the “between pathway model” in the *C. elegans* interactome (Lehner et al., 2006; Lehner, 2007). It was suggested that this difference might come from experimental screening methodologies employed to generate the GI interactomes in different organisms (Lehner, 2007). While in yeast most of the mutations used to disrupt genes are null, in *C. elegans*, they are mainly hypomorphic. The highest number of “within pathway” interactions in *C. elegans* when compared to yeast may then be explained by the fact that hypomorphic alterations of genes functioning within the same protein complex or signaling pathway, may lead to a significant aggravation of the phenotype (synthetic interaction) while this would not be the case for null mutations (Lehner, 2007). Also, we cannot exclude the possibility that this difference might come from the intrinsic difference existing between unicellular and multicellular organisms. “Within and between-pathway models” have not been used directly to predict novel GIs in the nematode.

While it is clear that signaling pathways are enriched in molecular interaction modules, it is important to notice the potential ambiguity created by the denomination of GIs occurring between dense molecular interaction modules as “between pathways” interactions. To the best of our knowledge, it has not been clearly proved that two densely connected molecular networks may not participate to the same signaling pathway – defined as a cascade of molecular events controlling a biological function. This possibility is supported by the fact that a high number of “pathways”/molecular interaction modules defined by Kelley and Ideker (2005) as well as Ulitsky and Shamir (2007), are very small (Ma et al., 2008). Consequently, we cannot exclude the possibility that some “between pathways/molecular modules” interactions may actually occur within signaling or metabolic pathways. This taken into consideration, the fact that most GIs in yeast occurs between molecular modules and presumably pathways constitutes a golden avenue to identify compensatory pathways responsible for the cellular homeostasis and development of resistance to therapeutic agents (Tucker and Fields, 2003; Szappanos et al., 2011). This hypothesis was validated experimentally using, for example, the Cdc14 early anaphase release (FEAR) and the mitotic exit network (MEN), two parallel pathways required for the release of the essential protein phosphatase Cdc14p from nucleolus during yeast cell cycle (Stegmeier et al., 2002).

Other approaches were used to study the modularity of GI networks. The decomposition of these networks using a biclustering technic recalled the idea of congruence. This technic was used to clusters groups of genes based on their GI profiles. However, in addition to clustering, biclustering helped the identification of two kinds of motif within the GI network: bicliques and q-cliques. This decomposition of the GI networks in absence of any integration of molecular networks gave also a bright new perspective to the within/between pathway models (Bellay et al., 2011a). In this study, the between pathway model implies that GIs occurs in “bicliques” – defined as biclusters in which the query genes (first cluster of genes) and the array genes (set of genes interacting with the query genes) do not overlap (**Figure 3E**). Following the same reasoning, within pathway interactions occur in “cliques/quasi-cliques/q-cliques” – defined as biclusters in which query and array genes have significant overlap (**Figure 3D**; Bellay et al., 2011a). Interestingly, both positive and negative interactions were mainly found in bicliques (Bellay et al., 2011a), similarly to what was shown using the canonical “between pathway” model (Costanzo et al., 2011). In addition, negative q-cliques – q-cliques composed of negative interactions – which corresponded to only 9% of negative biclusters (versus 91% of negative bicliques), did not appear to represent single protein complexes or pathways (Bellay et al., 2011a). This constitutes a major difference with the canonical “within pathway” model defined by the overlap of genetic and molecular modules (Kelley and Ideker, 2005). The genes found in negative q-cliques were found to be expressed in a coordinated manner and to be enriched for chromosome segregation and cell cycle processes (Bellay et al., 2011a). Bellay et al. (2011a) suggested that this particular functional enrichment might arise due to general sensitivity to perturbation in fragile systems such as cell division.

Altogether, these studies support the idea that different techniques used to decompose GI networks help revealing different categories of GIs. They suggest that predictive tools developed based on any of these models (the canonical “within /between pathway” model or the “biclique/q-clique” model) may be complementary to models built on the other one. The functional bias observed for different GI modules also suggests that predictive tools may gain in performance if they specifically target GIs associated to a subset of biological functions alongside homogenous particularities with respect to GI network modularity.

Network decompositions using biclustering techniques also help to provide critical information on duplicated genes (Bellay et al., 2011a). Duplicate genes were previously shown to display negative GIs with each other and exhibit fewer GIs than other genes because they tend to buffer one another functionally (VanderSluis et al., 2010). They were also shown to exhibit numerous unique GIs, suggesting that duplicated genes are functionally redundant but have divergent roles (Ihmels et al., 2007; VanderSluis et al., 2010). While, we would expect duplicated genes to be part of the isolated group of GIs within the biclustering array, a significant amount of them were fund to exhibit negative GIs with each other as part of larger modular structures (biclusters; Bellay et al., 2011a). Interestingly, this subgroup of duplicates was significantly more divergent in terms of sequence identity. It was suggested by Bellay et al. (2011a) that duplicates with a high degree of functional similarity specifically compensate for the loss of one another (isolated GIs in biclustering array), while in the second case, they appeared to have diverged into entirely different functional modules with compensatory properties (GIs being part of large biclusters). This study opens the door to predictive avenues that consider using protein sequence homology to identify compensatory genes and modules.

### EXPLOITING RELATIONSHIPS BETWEEN NETWORKS AT DIFFERENT ABSTRACTION LEVELS

Networks at different abstraction levels were used to infer GIs in yeast and *C. elegans* as detailed in **Table 1** and below. These studies also brought a deeper understanding of the molecular basis of GIs (Avery and Wasserman, 1992; Guarente, 1993; Thomas, 1993).

Genetic interaction in yeast, *C. elegans* and in human, were significantly more abundant between genes sharing mutant phenotypes (abstraction level VI) or gene ontology (GO) annotations (level V), and between genes encoding proteins in the same subcellular localization (level V) and/or within the same protein complex (level III) or pathway (level IV; Lee et al., 2004, 2008; Tong et al., 2004; Kelley and Ideker, 2005; Lin et al., 2010). In agreement with the general idea that synthetic GIs may occur between genes with redundant functions, the SSL yeast network was also found to be enriched in gene pairs encoding homologous proteins (level I).

A link between two genes or their protein products within networks located at different levels of abstraction is then informative of a potential GI. An important class of predictive methodologies used these diverse sources of data to discriminate interacting from non-interacting genes. The first of these studies used decision tree learning to integrate various types of data along with a “2hop” network topology assessment for various genomic relationships (**Table 1**; Wong et al., 2004). The “2hop” method considers gene pairs linked to a common partner by a functional relationship (e.g., physical interaction and sequence homology) to be informative of a potential SSL interaction between them in yeast. In total, 123 functional relationships (26 “major” categories) were used (Wong et al., 2004). The most powerfull predictive informations were selected using a Bayesian information criterion (BIC; similar to the Akaike information criterion, AIC).

For multicellular organisms, Zhong and Sternberg (2006) integrated multiple types of data from yeast, fly and nematodes to predict 18,183 GIs in the nematode *C. elegans *(**Table 1**). Here, a logistic regression was used to integrate features (or “attributes”) defined as the relative weight of a single type of data according to its predictive power. The positive set of elements used to train the model consisted in 1,816 validated GIs and 2,878 PPIs while negative examples were made of 3,296 paired *cis* markers. These makers are used in genetic mapping experiments and are assumed to have less probability of interacting together than pairs of genes randomly picked from the genome. The utilization of yeast/fly data to obtain greater genome coverage for a multitude of data sources appears to positively contribute to the predictive power of the developed tool (Zhong and Sternberg, 2006). We will discuss the limitation brought by data from other organisms in the following chapter considering evolutionary conservation of PPI and GI networks. In this study, the predictive interaction network was submitted to experimental validation using as bait *let-60*/Ras and *itr-1*/ITPR (two human disease-related genes) with a high success rate – 44 and 60% of true positive predictions respectively (Zhong and Sternberg, 2006). Although it is still unpublished, a new version of Zhong and Sternberg (2006) predictor, called “GeneOrienteer,” is available online (geneorienteer.org). This model employs a naïve Bayes classifier and integrates more than 20 features to predict GIs in several species.

Another approach, developed by Chipman and Singh (2009) , used a random walks algorithm to calculates the topological similarity of two genes in many types of biological networks, including genetic and physical interactions, co-expression and GO annotation networks, for both *S. cerevisiae* and *C. elegans* (**Table 1**). This topological similarity is then used to predict negative GIs. In this study, the decision tree classifier was shown to outperform the logistic regression classifier (Chipman and Singh, 2009). The good performances of this approach, tested using cross-validation technics, was unfortunately not supported by any experimental validations.

Other studies using the likelihood scoring of gene pairs for the prediction of GIs in the nematode *C. elegans* were generated soon after (**Table 1**; Lee et al., 2010a,b). The first approach, called “WormNet,” is used to infer the shared function of two genes, which is also indicative of a possible GI (Lee et al., 2008). This model was trained on thousands of gene pairs sharing GO annotations. A second version of this model, called ”WormNet2,” employs a weighted sum instead of a naïve Bayes classifier and integrates many “updated” features derived from log likelihood scores of various functional data (Lee et al., 2010b). Contrarily to Zhong and Sternberg (2006) methodology where functional data are more intuitive (e.g., co-expression of genes), WormNet2 included some “less-common” types of data (e.g., co-citation of gene names) as features to infer shared functions (Lee et al., 2010b). Although they did not use any feature selection methodology (e.g., BIC or AIC), several examples of resulting predicted interactions by the weighted sum model showed that most features contributed to the final scores. They also succeeded in validating several GI for three signaling genes via RNAi screening but the validation success rate for individual genes appears to be low ranged from only 4% to a maximum of 15% achieved for the gene *vab*-1 (Lee et al., 2010b).

Considering the environment of genes/proteins in networks at different level of abstractions, we built an additional model: “GIFinder” (**Table 1**; Lee et al., 2010a). This tool used logistic regression and six features to predict GIs with a positive training set composed uniquely of validated GIs. This model also used novel attributes that consider the enrichment of phenotypic features in the co-expression/physical network environment of a gene. This kind of attribute integrates data from three abstraction levels (level II, III, and VI) to assess whether two genes may be part of the same functional module instead of relying only on evidences of direct interactions. These attributes also reduced the negative effect of using biological datasets with poor genome coverage and were shown to highly contribute to the overall performance of the predictor (Lee et al., 2010a). This approach would be appropriate when trying to integrate sparse data such as tissue expression profiles and subcellular localization, to other datasets with high genome coverage such as expression data. Experimental validations of predicted GIs for *gdi-1*/GDI1 – a Rho GTPase regulator associated with non-syndromic forms of mental retardation in human – supported the idea that such methodology could be useful to identify therapeutic targets for monogenic diseases from predictive GI networks of lower organisms (Lee et al., 2010a). With a success rate of at least 42%, the performance in experimental validations was comparable to that of similar approaches.

Recently, Hoehndorf et al. (2013) created a predictor of GIs for 4 different species by inferring the function of many genes using semantic similarity measurements of phenotypes and GO annotations. The semantic similarity – a measure of the distance or relatedness between two terms – was done using the Jaccard index. Unfortunately, the GIs obtained from their inferred gene functions were not validated experimentally. This later methodologies exploit only biological information located at the highest level of abstraction (level V and VI). We expect that this methodology – ignoring co-expression and molecular interaction levels – would then be able to predict GIs occurring between genes controlling a given biological process from distant environments (cell non-autonomous interactions). However, this possibility has not been investigated by the authors (Hoehndorf et al., 2013).

When trying to compare the relative performances of predictive tools, it is important to note, that while experimental validation of predictions highly contribute to the demonstration of the validity of the method, the heterogeneity of link density within the GI network and the experimental methods used to validate the interactions may highly influence the success rate of the validation. Therefore, it is extremely difficult to compare the relative performance of individual methods just by comparing the success rate of validation experiments, using one or two genes as bait, and different validation methods (mutant and RNAi, mutant and double mutant, or RNAi and double RNAi).

**FIGURE 4:**
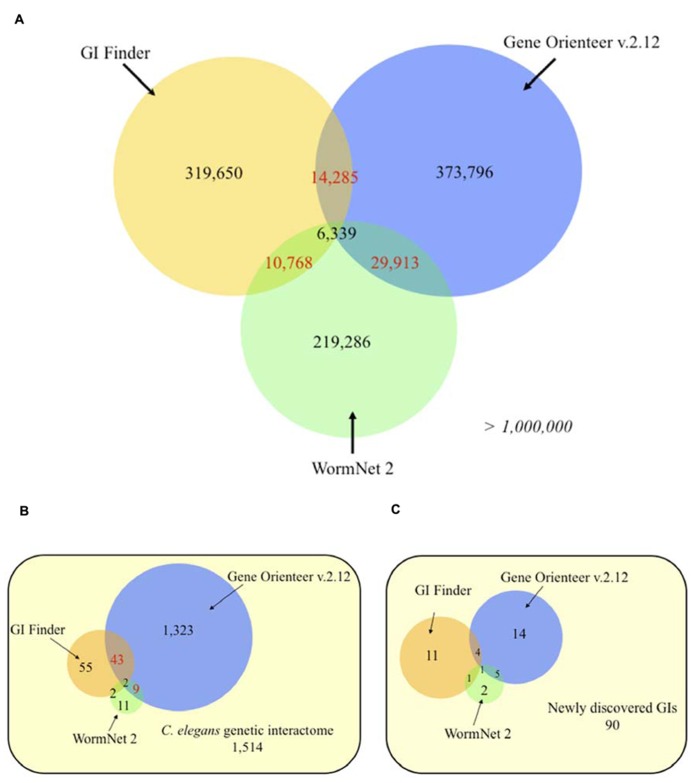
**Venn diagrams of *C. elegans* predicted genetic interactions from three different approaches.** **(A)** Genome-wide predictions. **(B)** Experimentally validated genetic interactions taken from Lee et al. (2010a). **(C)** Experimentally validated genetic interactions (GIs) collected from recent studies (2009–2012). Numbers in red indicates statistically significant overlaps (*P* < 0.05), evaluated using an exact hypergeometric probability test. The selected score thresholds used to predict GIs yield the same false positive rate (FPR) for all three predictors. Each FPR was evaluated using a negative set consisting of 10,000 random gene pairs free of any gene present in validated interactions. Predicted GIs or functional links, for GeneOrienteer and WormNet respectively, were downloaded in October 2010.

To assess how different integration designs impact the prediction of GIs for a given organism, we compared the predictions obtained for GeneOrienteerv2.12, GIFinder and WormNet2. Interestingly, these predictors appear to be highly complementary with more than 90% of predicted interactions by the three models being unique – i.e., predicted by only one approach (**Figure 4A**). This suggests that these three predictors capture different areas of the GI interactome covered by sets of experimentally identified GIs leaving more than 57% of it untouched (**Figure 4B**). GeneOrienteerv2.12 performed extremely well when tested on a set of 1,514 GIs obtained from interaction databases. This set of GIs, being used as a predictive feature or training sets in GIFinder and GeneOrienteerv2.12 (see “geneorienteer.org”; Lee et al., 2010a), we tested the three models on a set of recently published interactions (curated manually and absent from the databases) and observed a significant reduction in the performance of GeneOrienteer when compared to the two other models (**Figure 4C**). The deprived overlaps of predictions generated using the three predictors could be explained by the different integration methodologies used to generate the predictors (naïve Bayes classifier vs. linear regression) or by the different training sets used. The major difference of GIFinder when compared to others tools comes from the utilization of validated GIs as the only positive training examples as opposed to the two other ones that also employed physical interactions or functional annotations (Zhong and Sternberg, 2006; Lee et al., 2010a,b). While PPI and GI networks may have some overlap (some interactions occurring within protein modules), training a model using PPIs as a positive training set may bias the model toward within protein module GIs. Similar reasoning would be also valid for functional annotations. While functional annotations, such as GO annotations, are enriched between interacting genes, a large number of GIs are expected to occur between genes with different functions as discussed earlier. Interestingly, and as discussed in the following chapter, within protein module and within biological process GI appear to be more conserved that between modules or process GIs. We may then postulate that the bias induced through training the models using PPIs and GO annotations may increase the rate of evolutionary conserved interactions in the predictions. This taken into consideration, the fact that the training sets, constituted by the union of GIs and PPIs and/or pairs of genes with similar functions, is larger than validated sets of GIs only may improve the performance of predictive models using machine-learning techniques (Babyak, 2004).

While the existence of an edge between two genes/proteins in a network at a given level of abstraction is now confirmed as a useful information to infer a missing edge between these two genes/proteins at another level, it is important to realize that the conservation of links between two genes/proteins in different networks is a relatively rare event. For example, approximately 1% of SSL pairs (0.4% of negative and 0.5% of positive GIs in E-MAP datasets) codes for physically linked proteins (conservation of links between networks at levels III and IV) and 1% for homologous proteins (conservation of links between networks at levels I and IV; Tong et al., 2004; Costanzo et al., 2010). Cumulating these evidences of direct links between genes and proteins may increase the sensitivity of predictive tools for GIs. Considering only these direct links may also contribute to their relative poor performances. These tools would then gain in performance if integrating attributes that consider the environment of the genes in networks and the network modularity as shown by GIFinder (Lee et al., 2010a).

### CONSIDERING EVOLUTION OF PROTEIN–PROTEIN AND GENETIC INTERACTION NETWORKS

Several tools used data from evolutionary distant species to predict GIs. The evolutionary conservation of these data along with the structure of interaction networks between species is then of a critical interest when considering using this information to design a powerful predictive tool. In addition, while GI interactomes are extensively mapped in certain organisms such as yeast, the utilization of these networks to predict GIs in higher organisms mainly depends on the evolutionary conservation of GIs and of the GI network structure.

Genetic interaction are known to play a critical role in evolutionary processes (Yukilevich et al., 2008; Stern and Orgogozo, 2009). In opposition to what was initially thought, all genes are not equal in the eyes of evolution, and evolutionarily relevant mutations tend to accumulate in hotspot genes at specific positions of these genes (Stern and Orgogozo, 2009). A mutation in a gene, having a high number of GI partners, would not be advantageous in a context of adaptive evolution since it will increase the phenotypic variance associated with this mutation and therefore, will cause an increased fitness fluctuation dependent on the genetic background (Stern and Orgogozo, 2009). In addition, mutations generating specific phenotypic changes are more likely to contribute to adaptive evolution than pleiotropic mutations altering several seemingly unrelated traits (Stern and Orgogozo, 2009). Genetic Hubs, being by definition connected to a large number of genes and highly enriched for pleiotropic and multifunctional genes (Costanzo et al., 2010), would then be less touched by mutations associated with adaptive evolution. As expected, GI-Hubs are highly evolutionary conserved (Bellay et al., 2011b) as are PPI-Hubs (Wuchty et al., 2006).

When considering PPIs, interactions within modules are conserved at a higher level than interactions occurring outside modules (Zinman et al., 2011). This suggests that there might be a much higher selective pressure to maintain interactions within a single module than between modules (Zinman et al., 2011). PPI networks from distant species were used in number of studies to predict GIs (**Table 1**; Zhong and Sternberg, 2006; Chipman and Singh, 2009; Lee et al., 2010a,b; Hoehndorf et al., 2013). These studies, however, did not discriminate dense modules of PPIs from non-modular interactions. Since within complex/modules PPIs were shown to be more conserved than extra-modular PPIs, it would be interesting to assess whether the utilization of modular components of PPI interactomes from distant species, instead of the complete interactome, would improve performances of predictive tools for GIs.

While the evolutionary conservation of PPI- and GI-Hubs, as well as PPIs within protein complexes/modules has been clearly established, the overall conservation of GIs between evolutionary distant species is still controversial. Comparison of the *S. cerevisiae* and *S. pombe* E-MAPs showed that negative and positive GIs of two yeast species, distant of approximately 400 million years, were significantly conserved (Sipiczki, 2000). Also, essentiality in genes appears to be highly conserved between the yeast and nematode (Kamath et al., 2003), the extent of the GI conservation between these organisms appears to be very low, and not reported as significant in all studies (Pan et al., 2004; Lehner, 2007; St Onge et al., 2007; Mani et al., 2008; Tischler et al., 2008). The difference in methodologies used to generate the GI networks between yeast and nematodes, the fact that some GIs in nematodes may not be cell autonomous because of its multi-cellularity and the poor genome coverage of *C. elegans* vs. yeast genetic interactomes may be part of the reasons behind the poor conservation of GIs observed between these organisms.

Since we expect the majority of GIs not to be conserved across species, GI-Hubs, on the other hand, appear to be well conserved throughout evolution (Lehner et al., 2006; Costanzo et al., 2010). Predicting genetic Hubs are of biological importance because of their tendency to influence fitness defects when they are individually mutated (Costanzo et al., 2010). Some high-end methodologies have been developed to predict GI degrees – the number of GIs involving a given gene – in the yeast, *S. cerevisiae* (Szappanos et al., 2011; Koch et al., 2012). The first study successfully predicts negative and positive interaction degrees for genes implicated in yeast metabolism (Szappanos et al., 2011). Using only SSL and positive GIs as training sets, they showed that only a small fraction of interacting genes shares the majority of the interactions in both empirical and *in silico* data. They also provided a mechanistic explanation for genetic “Hubs” in relation with their tendency to be multifunctional and found that the predicted negative interaction degree of a gene correlates with its multifunctionality (Szappanos et al., 2011). In another work, Koch et al. (2012) drove the analysis furthermore to predict the GI degrees of many genes in *S. cerevisiae* and also in the distantly related species *Schizosaccharomyces pombe*. They integrated 16 features; covering mRNA expressions, GO terms, PPIs and other functional data, via a decision-tree learning to predict GI degrees with only interacting genes as training sets. Among some interesting findings, they confirmed the general consensus that the GI network structure is conserved across species (Koch et al., 2012). In fact, they found retaining high conservation of GI degrees between *S. cerevisiae* and *S. pombe* for specific genes sharing a significant amount of functional information. It would be extremely interesting to carry on such study to assess whether, despite the poor conservation of GIs between yeast and nematodes, the GI network structures may also be conserved between the two organisms.

As the conservation of GI-degrees, conservation of GIs between *S. cerevisia* and *S. pombe* was significantly increased when the analysis was restricted to genes that shared the same functional annotations and when the analysis was restricted to pairs of genes coding for interacting proteins (Roguev et al., 2008). This indicates that GIs between two genes is more evolutionary conserved if these two genes are also linked in networks located at lower and higher abstraction levels. Several studies also suggested that both positive and negative GIs within functional modules (protein complexes, gene belonging to the same biological process) are significantly more conserved between *S. cerevisiae* and *S. Pombe*, than wiring between these modules (Dixon et al., 2008; Roguev et al., 2008; Ryan et al., 2012). This suggests that not only the dependencies, but also the buffering relationships within complexes are highly conserved (Ryan et al., 2012).

While the conservation of GIs between functional modules/biological processes appears to be limited, the overall number of GIs between biological processes appears to be highly retained (Ryan et al., 2012). For example, while a significantly high number of GIs links genes controlling chromatin/transcription and those controlling mitosis and chromosome segregation in distant species, the level of conservation for individual interactions between these processes remains low (Ryan et al., 2012). This suggests that, although there is flexibility at the level of individual GIs and consequently significant rewiring between functional modules/processes in distant species, there may exist a biological selective pressure and requirements for the conservation of a high of low linking strength between particular processes (Ryan et al., 2012). Importantly, biological processes interacting with a larger amount of biological processes than expected – called here “process-Hubs” – suggest that these processes are important for mediating cross-process connections in genetic networks of several organisms (Lehner et al., 2006; Costanzo et al., 2010). For example, process-hubs such as chromatin/transcription, secretion and membrane trafficking, have been identified in *S. cerevisiae* (Costanzo et al., 2010) and *C. elegans* (Lehner et al., 2006). Conversely, some processes, such as amino acid metabolism and trans-membrane transport, have very few GIs linking them to other processes, suggesting a high degree of functional independency among these modules with less impact on other cellular processes than process-Hubs (Ryan et al., 2012).

Altogether, these data suggest that the conservation of the overall structure of GI networks still needs further characterization in distant organisms to identify the selective pressure applied on GI networks, not necessarily at the level of individual genes, but at the level of functional modules. Conclusions from such studies would bring important information that could be exploited in order to use GI networks from lower organisms to predict GIs in higher ones.

## CONCLUSION AND PERSPECTIVES

Mapping of GI networks and extensive study of their structures, conservation in different species and relationships with other functional and molecular interaction networks has already provided us with a better understanding of the biological robustness and phenotypical manifestation of genomic codes. Some of these pieces of information have also been exploited to generate predictors for GIs as detailed in this review. However, to date, these tools show limited performances and gave predictions, for example in *C. elegans,* for less than 50% of the expected GI interactome. These studies also opened some paths that could be followed to improve predictive tools for GIs.

**FIGURE 5:**
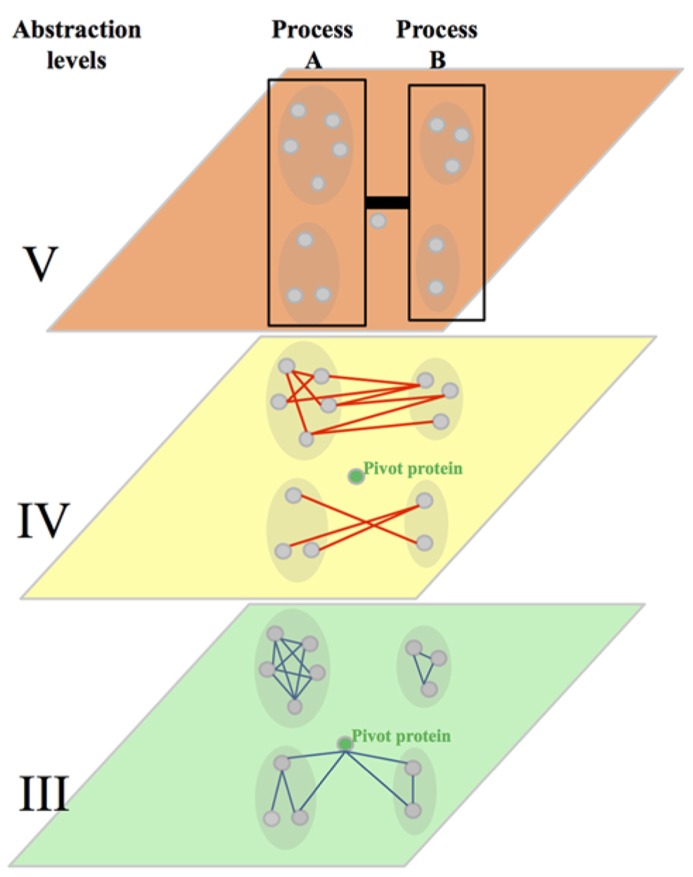
**Integration of the abstraction level III, IV, and V.** Abstraction level III shows protein–protein interactions (PPIs, blue edges) within highly connected protein interaction modules. It represents also a pivot proteins highly connected with proteins of two dense modules. The abstraction level IV shows the connection of dense protein modules through genetic interactions (GIs, red edges, between pathway model). It shows also the approximate rate of within pathway and between pathways GIs observed in yeast. The level V shows the clustering of dense modules in biological processes and the link brought by GIs between these processes. The strength of that link is more evolutionary conserved than individual GIs at the abstraction level IV.

The first path suggests that tools should consider GIs in their structural context instead of considering them in isolation. This comes from several observations. The first one showed that similarity of GI profiles of two genes is more indicative of a co-functionality (sharing GO annotations, involvement in the same protein complex, etc.) than a direct GI between these genes. This comes along with the other observations that – irrespective of the method used to decompose the genetic interactome into modules – GI tends to segregate into two categories following either a “within-“or a “between-pathway” model (**Figure 5**). These two kinds of GIs, based on structural properties of the network, have also different particularities. The “between-pathway” GIs tend to be less evolutionary conserved than the “within-pathway” GIs. Similarly, at a lower level of abstraction, “between protein modules” PPIs tend to be less conserved than “within protein modules” PPIs. Overall, these data suggests that “within and between pathways” GIs may have to be assessed using different approaches. This also suggests that data used to predict GIs, such as PPIs, may also have to be considered in their modular context.

The second path of improvement for predicting GIs consists in considering GIs from a higher level of abstraction when attempting to predict GIs using data from distant species. This comes from the observation that the overall level of GIs between biological processes appears to be much more conserved between distant species than independent GIs between genes involved in different processes (**Figure 5**). Considering GIs at the level of the biological processes (abstraction level V) instead of individual genes (abstraction level IV), may then significantly improve our ability to accurately predict functional relationships between genes and group of genes. Such approach may also open exciting opportunities. Studying the monochromaticity of GI modules also showed that the monochromatic within and between pathways interactions were biologically biased. This suggests that biological processes have either compensating or synergistic relationships one with another, but also that many components of a given biological process have predominantly either compensating or synergistic relationships. These data suggest that considering GIs from a higher level of abstraction may also be a good avenue to specifically identify synergistic and compensating/antagonistic relationships between functional biological modules. This avenue is particularly attractive when considering the need of such predictive tools in translational research and more particularly when trying to identify compensatory mechanisms leading to therapeutic drug resistance.

The last proposed path to improve GI predictions, in particular in higher organisms, is to try to better understand the structural differences that may exist between lower/unicellular and higher organisms. The fact that the within pathway model may be prevalent over the between pathway model in *C. elegans,* as opposed to yeast, need to be confirmed and the reason why this trend might be different in several organisms needs to be explained. In conclusion, while an extensive characterization of genetic networks in yeast has brought precious information about the still mysterious genetic interactome, its apparent plasticity requires similar studies to be done in higher organisms. These studies would then open the door to the design of well-informed and highly performing predictors for GIs in higher organisms such as human.

## Conflict of Interest Statement

The authors declare that the research was conducted in the absence of any commercial or financial relationships that could be construed as a potential conflict of interest.
